# 瘦素系统与非小细胞肺癌关系的研究进展

**DOI:** 10.3779/j.issn.1009-3419.2014.04.10

**Published:** 2014-04-20

**Authors:** 腾飞 韦, 龙坤 卢, 茜 沈, 超平 方

**Affiliations:** 200433 上海，第二军医大学附属长海医院实验诊断科 Department of Laboratory Diagnosis, Changhai Hospital, the Second Military Medical University, Shanghai 200433, China

**Keywords:** 瘦素, 瘦素受体, 肺肿瘤, Leptin, Leptin-receptor, Lung neoplasms

## Abstract

瘦素系统在肺部炎症反应、癌症发生发展等过程中发挥重要作用，但是其在肿瘤微环境中的作用机理、对肺癌的诊断价值仍不明晰。本文就瘦素系统与非小细胞肺癌之间的关系，从瘦素及其受体在循环和肿瘤组织中的表达变化、瘦素信号转导通路、瘦素与调节性T细胞的相互作用和瘦素及其受体的基因多态性等方面进行叙述，以期为非小细胞肺癌的诊治提供新方法。

## Abstract (en)

Leptin system plays an important role in lung inflammation and tumorigenesis, but the precise function in the tumormicrovironment and the prognosis value of the leptin system in lung cancer have not been fully elucidated. This review will focus on the relationship between leptin system and non-small cell lung cancer (NSCLC), in which special attentions will be paid on the expression of leption and its receptor (LepR) in peripheral blood and tumor tissues, leptin-related signal transduction pathways, the interaction between leption and regulatory T cells (Treg) and the gene polymorphisms of leptin and leptin receptor, and possibly provide new strategies for diagnosis and treatment of NSCLC.

最近，《*The World Cancer Report*》公开报道肺癌的发病率与死亡率居恶性肿瘤的首位并呈上升趋势；其5年总存活率仅16%，非小细胞肺癌（non-small cell lung cancer, NSCLC）占全部肺癌比例约为80%。同时，肥胖增加了肺癌发生的风险，随着体重指数增大，肺癌死亡率显著增高。

瘦素是于1994年发现的脂肪组织特异表达的多功能肽类激素，由肥胖基因编码并传递肥胖信号到大脑。它参与动物繁殖、造血和血管生成等生理过程^[1]^。瘦素除了在饮食调控、机体能量平衡等过程中发挥作用外，在癌症发生、发展和预后过程中也具有重要地位^[2]^。近期，瘦素及其受体与NSCLC之间的本质联系取得大量新进展，现就此作一简述。

## 1 瘦素和瘦素受体

人类瘦素的基因编码位于7q31.3，主要由白色脂肪组织产生并释放入血；在分泌入血过程中去掉N端分泌性信号肽，最终形成含146个氨基酸的16 kDa成熟瘦素^[1]^。作为细胞因子，瘦素mRNA也表达在下丘脑、肺、肝脏、肾脏、卵巢、胎盘、骨骼肌等组织中。瘦素受体（leptin receptor, LepR）主要生理功能是作为瘦素的结合蛋白或转运蛋白，使瘦素经过血脑屏障，到达作用位点以介导其功能，进而参与代谢、生殖、造血等病理生理过程和瘦素的自分泌调节。LepR是单次跨膜的细胞表面受体，结构上与Ⅰ型细胞因子受体家族相似，其6种拼接异构体（a、b、c、d、e、f）均具有相同的胞外段和跨膜区可延伸至胞膜内，但只有最重要的长型LepRb具有完整的胞内段，瘦素以此发挥其信号转导通路的最大效应。

## 2 瘦素、瘦素受体与NSCLC

胎儿和成人肺组织是瘦素产生和反应的靶器官，瘦素促进胎肺发育和肺的成熟，并维持肺功能稳态，参与成人肺泡细胞的生理性和病理性增殖过程^[1]^；而最新研究^[3]^发现，瘦素在决定肺功能最佳状态方面也具有重要作用。众所周知，体内瘦素/LepR系统作为内分泌腺可以促进良、恶性肿瘤上皮细胞内的血管生成，体外实验^[4]^发现，动物和人细胞系中的LepR的高表达促使肿瘤细胞增殖。近期，Ntikoudi等^[5]^对发表的有关瘦素与肺癌关系的文献报道进行了系统分析，结果发现，瘦素和脂联素是研究最多的，主要包括瘦素在肺癌或肺癌恶液质状况下与患者的营养状态、系统性炎症及评价预后的意义等。越来越多的文献证实瘦素系统在肺癌发生、进展和肺部肿瘤免疫应答中均发挥重要作用。

### 2.1 NSCLC患者循环和肿瘤组织中的瘦素及其受体表达水平与疾病发生和发展的关系

瘦素与肺癌的早期研究主要集中在血清瘦素含量浓度、肿瘤组织内瘦素表达水平与NSCLC的生成、病程进展间的关系，此方面研究数据较多，但是结果各异。早在2007年，Carpagnano等^[6]^研究发现，NSCLC患者的呼出气冷凝液、痰液、血液、尿液及支气管肺泡灌洗液中瘦素水平明显增加，故认为升高的瘦素在NSCLC的发生中发挥了重要作用。与此相反^[7]^，以未经任何治疗的肺癌患者为主要研究对象，发现肿瘤患者血清中瘦素水平比正常人明显降低，而脂联素等脂肪细胞产生的因子却升高；且瘦素水平越低，患者的生存时间越短。因此，作者认为，血清瘦素浓度可以为NSCLC患者提供一个预后指标^[7]^。然而也有临床研究发现，在进行性别和体重指数（body mass index, BMI）校正后，NSCLC患者与健康对照组之间血清瘦素水平无明显差异^[8]^。出现恶病质的NSCLC患者的血清瘦素水平比非恶病质和正常人明显下降，血清瘦素水平低的患者其总体生存率降低，可以把瘦素水平的下降作为肺功能改善与否的预警信号^[9]^。与此相互印证的是，在其它晚期肿瘤患者如前列腺癌中也发现血清瘦素水平降低。Werynska等^[10]^认为，血清中的高瘦素水平与早期肺癌发病有关，持续暴露于高浓度瘦素下可以促进癌症发生（瘦素浓度升高是促肿瘤事件），而发展到肺癌晚期，由于恶病质、食欲下降等原因，患者脂肪含量和BMI均降低，进而导致瘦素生成减少。最近文献^[11]^报道，禁食可诱导小鼠形成低瘦素血症，这与肺癌晚期食欲下降的原理相同。由于肺癌患者下丘脑对瘦素的反馈调节机制并没有因为肿瘤进展所导致的厌食和恶病质而受到影响，但晚期肺癌患者血清瘦素浓度较早期患者及正常人明显降低，可能与晚期肺癌恶病质导致患者体脂水平低下对瘦素分泌的反馈作用有关^[12]^。晚期癌症患者出现的恶病质是应激型的营养不良，为多因素所致，并非由于瘦素生成的失调^[8]^。循环中瘦素水平的降低应该是恶病质的结局，而不是起因^[8]^。上述的研究结果表明，NSCLC患者的血清瘦素水平受患者的疾病进展、病理分期、是否出现恶液质等因素的影响和调控。同时，造成结果的差异也与临床研究数据是否严格进行了BMI、性别等的校正有关。

肺癌组织中瘦素主要表达在肿瘤细胞的胞浆中，LepR则在细胞膜上和胞浆中均有表达^[13]^。Xu等^[14]^用免疫组化实验证实，71%的NSCLC肿瘤组织中瘦素表达阳性，而正常肺组织仅25%阳性；LepR的表达在肿瘤组织的表达也明显高于正常肺组织。单变量生存分析表明，瘦素表达与肿瘤TNM分期相关，影响生存时间；多变量分析提示瘦素表达是NSCLC独立的危险因子^[14]^。与肿瘤组织瘦素和LepR均不表达的^[14]^相比，瘦素及其受体共表达的NSCLC^[14]^临床预后很差，同时也表明功能性瘦素信号转导相比瘦素本身来说，是更重要的预后决定因素^[4]^。

### 2.2 瘦素及其受体在NSCLC发生和发展中的可能作用机制

众所周知，瘦素可以促进血管内皮细胞增殖和血管重塑，当其在肺肿瘤组织中表达时可以使血管内皮细胞生长因子表达上调^[15]^；瘦素受体介导的下游信号可以使内皮细胞的瘦素和LepR基因表达上调^[16]^。

#### 2.2.1 瘦素通过自身作用直接参与NSCLC疾病进展和肿瘤转移

瘦素介导肺癌形成过程中的免疫炎性机制，持续存在的炎症反应导致上皮细胞恶变。Shen等^[17]^认为瘦素与外周血单个核细胞表面的LepR结合，诱导促炎细胞因子并抵抗凋亡，可以促进肺癌的免疫逃逸，最终导致NSCLC患者预后不良。瘦素是通过什么机制导致NSCLC细胞抵抗凋亡？Wang的研究组^[18]^以A549肺腺癌细胞株研究了瘦素的抗凋亡机制，发现瘦素可以阻断内质网应激所介导的凋亡，促进肿瘤细胞增殖；这种阻断作用是通过抑制CCAAT/增强子结合蛋白的激活，从而降低了磷酸化的蛋白激酶R样内质网激酶（protein kinase R-like ER kinase, PERK）和活化转录因子6（activating transcription factor 6, ATF6）的信号通路活性所引起。

瘦素刺激免疫细胞活化、炎性细胞因子生成的生物学效应，主要通过激活与免疫炎性因子生成有关的关键信号通路，如细胞外调节蛋白激酶（extracellular signal-regulated kinase, ERK）、蛋白激酶B（protein kinase B, PKB/Akt）、促分裂素原活化蛋白激酶（mitogen-activated protein kinase, MAPK）和哺乳动物雷帕霉素靶蛋白（mammalian target of rapamycin, mTOR）等信号通路^[17]^。同时，瘦素又可通过MAPK、ERK和Akt/mTOR等信号通路的活化反过来进一步促进自身基因表达^[15, 17]^。在NSCLC^[14]^肿瘤组织中，Akt、mTOR等磷酸化表达明显增高，血管内皮生长因子、*Akt*和*mTOR*基因的mRNA表达水平也均较癌旁肺组织明显升高^[19]^。Pathak等^[15]^研究发现，磷脂酰肌醇3激酶（phosphatidylinositol 3 kinase, PI3K）/Akt/mTOR信号通路在NSCLC患者中呈活化状态，瘦素激活mTOR通路的重要分子P13K，而Akt是P13K下游的作用靶点，mTOR为Akt的一个底物。此信号通路活化可以调节肺癌细胞多种酪氨酸激酶受体的表达，促进癌细胞的增殖和存活，在肺癌的发生和发展中发挥重要作用^[15]^。

#### 2.2.2 瘦素系统通过调控Treg细胞间接参与NSCLC疾病进展和肿瘤转移

调节性T细胞（regulatory T cell, Treg）是一类具有免疫抑制作用的CD4^+^ T细胞，以主动方式参与机体免疫应答/免疫耐受的调控。Treg细胞介导的免疫抑制是肿瘤免疫逃逸的重要机制，也是肿瘤免疫治疗的主要障碍。多篇文献^[20, 21]^证实，NSCLC患者外周血中Treg细胞比例明显高于正常人，肿瘤组织浸润的Treg细胞比例也明显高于炎性肺组织；腺癌组织中Treg细胞的浸润比例明显高于鳞癌，并随肿瘤分期增加而增加，Treg细胞比例越高其预后越差。Treg细胞比例与NSCLC的病理状态和肿瘤负荷相关^[22]^，手术切除肺癌组织后外周血Treg细胞比例明显降低^[20]^。肺腺癌肿瘤相关成纤维细胞可以产生较多的转化生长因子β和血管内皮生长因子，诱导Treg细胞的生成，形成了促肿瘤生长的微环境^[22]^。在小鼠肺肿瘤形成的早期，使用抗-CD25单克隆抗体进行治疗可有效清除Treg细胞，提高CD8^+^ T细胞表达颗粒酶A、颗粒酶B、穿孔素和干扰素γ的水平，增强CD8^+^ T细胞介导的对肿瘤细胞的杀伤作用，控制或阻断肿瘤进展。在肿瘤晚期，联合卡铂化疗的肿瘤鼠存活率比单一清除Treg细胞明显升高，表明Treg细胞清除联合细胞毒药物化疗可作为晚期NSCLC的有效治疗方法^[26]^。

Treg细胞与瘦素之间存在什么样的关系？研究证实瘦素系统对Treg细胞的增殖和功能有明显的抑制作用，控制着机体的自我耐受。瘦素基因敲除（ob/ob）小鼠和瘦素受体基因敲除（db/db）小鼠循环Treg细胞数量均明显增加^[23]^。最新文献^[11]^报道，禁食可以诱导小鼠形成低瘦素血症，低瘦素血症又引起Treg细胞增殖和功能增强；通过给予瘦素可逆转这个反应。因此，在瘦素系统基础上干预免疫应答，可以通过抑制Treg细胞的数量和活性来控制癌症的发展。

Treg细胞需要独特的能量代谢途径以支持其特异性的功能^[24]^。mTOR是一种保守的289 kDa丝/苏氨酸蛋白激酶，能够整合来自营养、能量和生长因子的环境信号，调控细胞生长、增殖和分化^[24]^。瘦素-mTOR轴可以把细胞的能量状态与Treg细胞代谢相关的信号整合到一起，通过激活mTOR信号通路来调控Treg细胞的增殖能力，促使能量协调和Treg细胞的动态平衡^[25]^。新鲜分离的Treg细胞可以产生瘦素，并且大量表达LepR^[24]^。Treg细胞产生的瘦素也可通过自分泌和旁分泌引起自身细胞内mTOR信号通路活化，继而限制自身增殖^[23]^。这样就在瘦素-mTOR-Treg细胞之间形成动态性的回路，使机体免疫调节达到平衡状态。

### 2.3 瘦素、瘦素受体的基因多态性与非小细胞肺癌

瘦素基因-5’端的单核苷酸多态性对瘦素的表达有影响。在瘦素基因（-2548 G/A）启动子区域上游的一个高表达的、功能性的同质多态性使NSCLC患者早期致病^[26]^。瘦素基因增强子的多态性导致瘦素表达增加者，其患NSCLC的风险增加3倍^[27]^。一种瘦素基因的功能性多态表型（AA基因型）与NSCLC患病风险有关（尤其对吸烟者而言）；经过年龄和性别校正后，证实AA基因型是肺癌的独立风险因子^[27]^。

人类*LepR*基因的多态性与多种肿瘤的发生相关。在NSCLC患者*LepR*基因Gln223Arg的等位基因频率和Arg/Arg基因表型均比正常对照明显增多。Arg/Arg基因表型的携带者对NSCLC具有明显高风险（OR3.1）；Arg/Arg携带者表现为较高的TNM分期，预后差^[13]^。研究者^[13]^认为LepR基因的Gln/223Arg单核苷酸多态性可作为NSCLC临床进展和预后的分子标志物。

## 3 问题与展望

在过去的几年里，瘦素系统在NSCLC发生和发展中作用的研究取得了长足的进步。然而，瘦素作为一种多功能激素，由于其复杂的多系统、多靶细胞、多信号传导通路的调控作用，要阐明瘦素系统在NSCLC发生和发展中的影响，并非易事。目前，很多实验结果是从肺癌细胞系或肥胖动物模型（ob/ob小鼠或db/db小鼠）获得的，不能完全代表人类肺癌复杂的病理状态，导致目前对瘦素在NSCLC发生和发展中作用和机理的认识仍较有限。因此，急需深入研究瘦素系统在NSCLC发生发展中的作用，为肺癌早预防、早诊断、早治疗提供新思路和新途径。肿瘤局部微环境中瘦素-mTOR轴对于Treg数量和功能发挥作用的机制目前尚未完全阐明，进一步探讨NSCLC患者肿瘤微环境中瘦素-mTOR轴对Treg细胞增殖活化和功能发挥的机理，有利于发现靶向Treg细胞、逆转肿瘤免疫逃逸、提高杀伤肿瘤效果的新治疗方法。
